# Seroprevalence, predictors and estimated incidence of maternal and neonatal Herpes Simplex Virus Type 2 infection in semi-urban women in Kilifi, Kenya

**DOI:** 10.1186/1471-2334-11-155

**Published:** 2011-05-31

**Authors:** Joyce U Nyiro, Eduard J Sanders, Caroline Ngetsa, Steve Wale, Ken Awuondo, Elizabeth Bukusi, Matthew A Price, Pauli N Amornkul, D James Nokes

**Affiliations:** 1 Centre for Geographic Medicine Research-Coast, Kenya Medical Research Institute, Kilifi, Kenya; 2 Centre for Clinical Vaccinology & Tropical Medicine, University of Oxford, Oxford, UK; 3 Kenya Medical Research Institute/ Centre for Microbiology Research, Nairobi, Kenya; 4 International AIDS Vaccine Initiative, New York, New York, USA; 5 School of Life Sciences, University of Warwick, Coventry, UK

## Abstract

**Background:**

Herpes Simplex Virus type 2 (HSV-2) has public health importance as a leading cause of genital ulcers, a co-factor in HIV-1 acquisition and transmission and as a cause of neonatal herpes infections. Little is known of its epidemiology and burden in Coastal Kenya.

**Methods:**

We screened plasma samples for HSV-2 infection from 826 women aged 15-34 years who participated in an HIV-1 survey in Kilifi in 2004. The sample comprised 563 women selected randomly from a demographic surveillance system (DSS) and 263 women who presented for voluntary counseling and testing (VCT). Predictors for HSV-2 seropositivity were determined using multivariate logistic regression. The incidence of HSV-2 infection and risk of neonatal herpes were estimated by a simple catalytic model fitted to age-seroprevalence data.

**Results:**

HSV-2 prevalence was 32% in the DSS recruits vs. 44% in the VCT recruits (P < 0.001), while, HIV-1 prevalence was 8% in the DSS recruits vs. 12% in the VCT recruits (P = 0.12). Independent risk factors for HSV-2 infection in all women were: older age (30-34 years; odds ratio (OR) 10.5, 95% confidence interval (CI): 5.2 - 21.0), recruitment from VCT (OR 1.5, 95% CI: 1.1 - 2.1), history of genital ulcers (OR 1.7, 95% CI: 1.2 - 2.3) and HIV infection (OR 2.7, 95% CI: 1.6-4.6). Education beyond primary (OR 0.7, 95% CI: 0.5 - 0.9) was inversely associated with HSV-2 infection. In the DSS sample, HSV-2 incidence was estimated at 4 cases (95% CI: 3.3 - 4.4) per 100 women per year, 17 cases (95% CI: 16-18) per 1,000 pregnancies per year and 33 neonatal cases (95% CI: 31-36) per 100,000 births per year.

**Conclusions:**

HSV-2 transmission is rapid following the onset of sexual activity and likely to result in a significant burden of genital ulcer disease. Nevertheless, the burden of neonatal HSV-2 can be predicted to be low. Educating young women about HSV-2 infection may help in reducing its burden in this semi-urban population.

## Background

Herpes Simplex Virus type 2 (HSV-2) infection among women of the general population worldwide is of considerable public health importance as a leading cause of genital ulcer disease [1,2], neonatal herpes infections [3-6] and due to its role in enhancing HIV-1 acquisition and transmission [7-9].

Establishing the burden of HSV-2 infection can be difficult because incident cases and reactivations are often missed clinically [6,10,11], and vertical transmission is a rare occurrence in populations with low HSV-2 prevalence [12-15]. About 80% of neonatal herpes infections arise from primary genital HSV-2 infection, acquired late in pregnancy [3,5,6,15], and can result in significant morbidity to the newborn child [4-6]. Reactivated HSV-2 infections are estimated to cause <5% of neonatal herpes transmission [5,15]. Neonatal herpes infections can also be caused by HSV-1 infection in about 20% of the instances [5,15]. In the USA, neonatal herpes incidence has been estimated as 1 case per 3200 live births in a study involving over 58000 live births born in the period 1982-1999 [3]. In Africa, there is no information on the proportion of pregnant women who acquire HSV during pregnancy or on the incidence or prevalence of neonatal HSV-2 infection[16].

Studies in the African continent have shown variation in HSV-2 prevalence across diverse populations of women, ranging from 22% among adults in Tanzania and 68% among adults in urban Kenya to 90% among commercial sex workers in Zaire [9,17]. In Western Kenya, an HIV survey among women aged 13-34 years conducted in 2003-4 revealed an HSV-2 prevalence of 53% [18]. A more recent National AIDS Indicator survey among general population women aged 15 - 64 years estimated HSV-2 prevalence of 42% but data on the possible impact of maternal transmission of HSV-2 were not provided in that survey [19]. Prevalence data, however, provide information on the proportion of individuals infected and allow for an estimation of HSV-2 incidence. Hence, vertical transmission can be inferred [3,14,15].

In this study we explore the prevalence of and predictors associated with HSV-2 seropositivity, the estimated rate of infection and the potential risk of vertical transmission using two sample sets from within a well-enumerated population of semi-urban women from coastal Kenya, namely, (i) a random sample of the adult women population and (ii) self-selected women attending for voluntary counselling and testing (VCT).

## Methods

Kilifi District is a largely rural district in Coastal Kenya, with an estimated population of 544,305 people (52% female)[20]. In the year 2000, the Kenya Medical Research Institute (KEMRI) - Wellcome Trust Research Programme defined and mapped a geographical area in Kilifi District for demographic surveillance and clinical and epidemiological research. The resultant Kilifi Demographic Surveillance System (DSS) monitors a population of around 240,000 residents [21]. Kilifi District has a birth rate of approximately 8,000 live births per year, perinatal mortality of 44 per 1000 live births [21] and about 4,000 women attend antenatal care at the district hospital per year [21]. Until 2003, in Kilifi, pregnant women were not routinely tested for HIV-1, and VCT services were not widely available. In 2004, a large HIV-1 survey was conducted at several semi-urban communities in and around Kilifi town and VCT services were strengthened.

In this study, we used data and serum samples of women aged 15-34 years who were participants of the 2004 HIV-1 survey and resided in Kilifi and Mtondia towns[21]. Kilifi and Mtondia areas are considered semi-urban, as they are not densely populated, have few paved roads and very few people have electricity.

Study participants were recruited (a) from the registers of the DSS by simple random sampling or (b) as they presented to a VCT-clinic supported by the study. The DSS participants were visited by local fieldworkers in their homes during daytime for recruitment into the HIV-1 study. Overall non-participation was approximately 30%, as has previously been reported when the DSS register is used for population sampling[22]. Non-participation was predominantly caused by women who were not present at the time of visiting the household. No records of refusal were kept for non-respondent women.

At recruitment, all volunteers were interviewed, counselled and educated about the HIV prevalence study prior to enrolment. Acceptance of HIV-1 testing was an enrolment criterion. All volunteers gave informed consent for storage of their blood samples and for more testing of those samples on other infections related to HIV. A survey questionnaire was administered through one on one interview for collection of data on socio-demographic characteristics, sexual exposure, medical history and knowledge of sexually transmitted disease (STD) / HIV-1 related infections. A medical history that focused on the past or present STDs was obtained. Medical care for prevalent sexually transmitted diseases was provided. Volunteers received pre- and post-HIV test counselling, and were tested for HIV-1 at the enrolment visit using two rapid test kits (Determine, Abbott Laboratories, Abbott Park, Illinois, USA; Unigold, Trinity Biotech plc, Bray, Ireland) in parallel. Discrepant rapid HIV-1 test results were resolved using an ELISA test (Genetic System HIV-1/2 plus O EIA, Bio-Rad Laboratories, Redmond, Washington, USA). Women who tested HIV-1 positive were offered a pregnancy test and referred for comprehensive HIV care, including Prevention of Mother to Child Transmission (PMCTC) services, as appropriate, at the District Hospital.

After testing for HIV-1, serum samples were stored for other tests related to HIV-1 infection. The Kenya National Ethical Review Committee approved this study.

### Determination of HSV-2 antibody status

Approximately 10 ml of blood was collected from each study participant, and the serum was stored at -70°C. HSV-2 specific antibody status was determined for serum samples using the HerpeSelect 2 ELISA IgG (Focus Diagnostics, Cypress, California, USA) according to the manufacturer's protocol [23] with the following exception. The protocol recommends a cut off index value of 1.1 to determine HSV-2 infection. Due to a lower specificity of the HerpeSelect test on serum samples from African populations, we used a cut off value of > 3.5 to determine HSV-2 prevalence and incidence estimates [24-26].

### Statistical analyses

#### (i) Analysis of predictors of HSV infection

We conducted the analyses using STATA version 10 (StataCorp, College Station, Texas, USA). Chi-squared and Fisher's exact tests were used to determine univariate associations with prevalent HSV-2 infection. Multivariate logistic regression was used to identify independent predictors of HSV-2 seropositive status. Variables were introduced sequentially into a multivariate model, beginning with those with the strongest univariate association (lowest p values) and including only those which provided a significantly improved fit to the data (a likelihood ratio test, LRT, P < 0.05). The correlation coefficient between two variables was used to determine whether to exclude a variable from analysis due to excessive colinearity (i.e., r^2 ^> 0.2).

#### (ii) The risk of primary maternal and neonatal HSV-2 infection

The incidence of maternal HSV-2 infection and risk of neonatal herpes were estimated using a simple catalytic model as described in [27]. In this model, the force of infection (i.e. per person incidence) was calculated by fitting age-stratified prevalence of specific antibodies to an exponential decay model by maximum likelihood. The assumption was made that seronegative status equates to the absence of HSV-2 infection (past or present), and that following infection there was no reversion to seronegativity. In other words we assumed the seronegative status is equated with being susceptible to infection. We further assumed that all women were seronegative at age 14 years and thereafter exposed to a force of infection (per year), λ, constant across all ages and unchanging over time (where λ > 0). Hence the proportion susceptible, *s (a*), at age *a *can be defined as(1)

where 14 <*a *< 35. The proportion seropositive at age *a*, *p(a)*, is therefore *1-s(a)*. We fit this model by maximum likelihood to our observed proportions remaining seronegative, *S(i)*, representing *r(i) *seronegative individuals out of *n(i) *sampled for each single year of volunteer age, *i *= 15-34, to identify the value of the force of infection best supported by the data and estimate the 95% confidence intervals [27].

We made the assumption that the force of infection acting on pregnant women is equivalent to that estimated from our seroprevalence survey. If we now assume that the childbearing age range is 15-44 years, with average proportion susceptible  derived from (1), and given a gestation period of 40 weeks, it follows that the average annual risk of primary maternal HSV-2 infections per pregnancy, *I_m_*, is(2)

which for small λ approximates to(3)

Assuming vertical HSV-2 infection only arises if a pregnant woman is shedding virus at the time of birth [6], that is, only for a primary or initial HSV-2 infection during the final 11 days of pregnancy [28], and that this transmission risk, *v*, is 50% [5,15], then from equation (3) the risk of neonatal HSV per pregnancy,*I_n_*, is(4)

The number of maternal infections per 1,000 pregnancies and neonatal infections per 100,000 live births can thus be defined from equations (3) and (4), respectively; the latter assuming there is no excess mortality attributable to HSV-2 infection.

## Results

The sample comprised 826 women of median age 24 years (Interquartile range (IQR): 15-34 years), of whom 563 (68%) were selected by random sampling from the DSS (median age of 24 years; IQR 21-29), and 263 (32%) were individuals presenting to the VCT-clinic (median age 24 years; IQR 21-28). Details of demographic characteristics of these women in the two groups (DSS and VCT participants) are given in (Table 1).

**Table 1:** Characteristics of participating women from Kilifi, Kenya

Characteristic	DSS^μ^	%		VCT^α^	%		Total	%		P*
**Sample size**	563			263			826			
**Age, years**										
Median (range)	24 (15-34)			24(18-34)			24(15-34)			0.536^
15-19	105	19		30	11		135	16		0.001
20-24	179	32		118	45		297	36		
25-29	142	25		67	25		209	25		
30-34	137	24		48	18		185	22		
**Marital status**										
Single	172	31		70	27		242	29		0.002
Married monogamous	314	56		129	49		443	54		
Married polygamous	36	6		26	10		62	8		
Widow/separated	41	7		38	14		79	10		
**Education**										
Completed primary	247	44		119	45		366	44		0.764
**Religion**										
Christian	325	58		183	70		508	62		0.005
Muslim	135	24		46	17		181	22		
Other	103	18		34	13		137	17		
**Ethnicity**										
Giriama	375	67		171	65		546	66		0.128
Chonyi	60	11		19	7		79	10		
Other	128	23		73	28		201	24		
**Condomuse sometimes**										
Yes	74/419	18		74/228	32		148/647	23		<0.001
**Casual partners in last year**										
Yes	66/428	15		49/158	31		115/586	20		<0.001
**Partners in last year**										
None	238/429	55		93/158	59		331/587	56		<0.001
One	162/429	38		39/158	25		201/587	34		
More than one	29/429	7		26/158	16		55/587	9		
**History genital ulcer**										
Yes	161	29		114	43		275	33		<0.001
**Genital ulcer now**										
Yes	56/479	12		45/258	17		101/737	14		0.033
**HIV status**										
Pos	46	8		31	12		77	9		0.122
**HSV-2 status (>3.5 cut off)**										
Pos	181	32		115	44		296	36		<0.001

^μ^DSS - Demographic Surveillance System

^α^VCT - Voluntary Counselling and Testing.

*P - Chi squared P value

The two sampling groups differed in their marital status and religion (Table 1). Women attending VCT, when compared to the random sample from the DSS, were more likely to have used condoms, to have had casual sexual relationships, had more sexual partners in the previous year, and also to have genital ulcers (either presently or ever). The two groups did not differ significantly in HIV-1 prevalence (12% in VCT vs. 8% in DSS, P = 0.12), ethnic composition, educational level attained, or age distribution. There was a higher seroprevalence of HSV-2 in VCT-women compared with DSS-women (44% (115/263) vs. 32% (181/563), P < 0.001). Of 77 HIV-1 positive women, 50 (65%) were HSV-2 positive, compared to 33% (246/749) of HIV-1 negative women (p = 0.001).

### Predictors of HSV-2 seropositivity

Significant associations from univariate analysis are detailed in Table 2. The probability of being HSV-2 seropositive was significantly higher in women who were of older age, were sampled from the VCT clinic, were ever married, were less educated, had causal partners in the last year, had ever had genital ulcers, or were HIV-1 antibody positive. Marital status was correlated (r^2^>0.2) with multiple factors (including educational status, HIV-1 status, religion, use of condoms and history of genital ulcers) and was dropped from further analysis. Factors shown to be independently associated with HSV-2 sero-positivity using multivariate logistic modelling are shown in Table 3. HSV-2 positive status was found to be associated with increasing age, recruitment from the VCT centre, history of genital ulcers and positive HIV-1 sero-status. Higher educational level was inversely associated with HSV-2 seropositivity. Separate multivariate models for DSS and VCT women were developed, which gave similar results to those of the full model, except that for DSS women the association with present genital ulcers was replaced with ever having had genital ulcers, and for VCT women the association with casual partners in the last year was lost. In the full model no factors significantly interacted with the association between study group (DSS vs. VCT) and HSV-2 status.

**Table 2:** Risk factors for HSV-2 in semi-urban women in Kilifi, Kenya: univariate analysis

	HSV2				95%CI		
Characteristic	pos	n	%	OR	LCL	UCL	P
**Age, years**							
15-19 (ref)	11	135	8.1				
20-24	98	297	33.0	5.6	2.9	10.8	<0.001
25-29	90	209	43.1	8.5	4.3	16.7	<0.001
30-34	97	185	52.4	12.4	6.3	24.5	<0.001
**Study group**							
DSS (ref)	181	563	32.1				
VCT	115	263	43.7	1.6	1.2	2.2	<0.001
**Marital status**							
Never married (ref)	42	242	17.4				
Married monogamous	173	443	39.1	3.1	2.1	4.5	<0.001
Married polygamous	33	62	53.2	5.4	3.0	9.9	<0.001
Ever married	48	79	60.8	7.4	4.2	12.9	<0.001
**Education**							
None or some primary (ref)	183	460	39.8				
Completed primary	113	366	30.9	0.7	0.5	0.9	<0.008
**Religion**							
Christian (ref)	160	508	31.5				
Muslim	75	181	41.4	1.5	1.1	2.2	0.016
Other	61	137	44.5	1.7	1.2	2.6	<0.005
**Ethnicity**							
Giriama (ref)	190	546	34.8				
Chonyi	26	79	32.9	0.9	0.6	1.5	0.742
Other	80	201	39.8	1.2	0.9	1.7	0.207
**Condom use**							
Never (ref)	209	499	41.9				
At least sometimes	50	148	33.8	0.7	0.5	1.0	0.078
**Casual partners in last year**							
No (ref)	179	471	38.0				
Yes	57	115	49.6	1.6	1.1	2.4	0.024
**Number of partners in last year**							
None (ref)	143	331	43.2				
One	68	201	33.8	0.7	0.5	1.0	0.033
More than one	26	55	47.3	1.2	0.7	2.1	0.573
**History genital ulcer**							
No (ref)	158	551	28.7				
Yes	138	275	50.2	2.5	1.9	3.4	<0.001
**Genital ulcer now**							
No (ref)	229	636	36.0				
Yes	61	101	60.4	2.8	1.8	4.5	<0.001
**HIV status**							
Negative (ref)	246	749	32.8				
Positive	50	77	64.9	3.8	2.3	6.2	<0.001

**Table 3:** Risk factors for HSV-2 in semi-urban women in Kilifi, Kenya: multivariate analysis (n = 826)

	95%CI			
Characteristic	OR^α^	LCL*	UCL*	P
**Age, years**				
15-19 (ref)				
20-24	4.7	2.4	9.2	<0.001
25-29	6.9	3.5	13.7	<0.001
30-34	10.5	5.2	21.0	<0.001
**Study group**				
DSS (ref)				
VCT	1.5	1.1	2.1	0.010
**Education**				
None or some primary (ref)				
Completed primary	0.68	0.50	0.94	0.018
**History of genital ulcer**				
No (ref)				
Yes	1.7	1.2	2.3	0.002
**HIV status**				
Negative (ref)				
Positive	2.7	1.6	4.6	<0.001

^α^OR, Odds Ratio,*LCL, UCL, Lower and upper 95% confidence interval, respectively

### Incidence estimates of maternal HSV-2 infection and estimated risk of neonatal herpes

**Figure 1:**
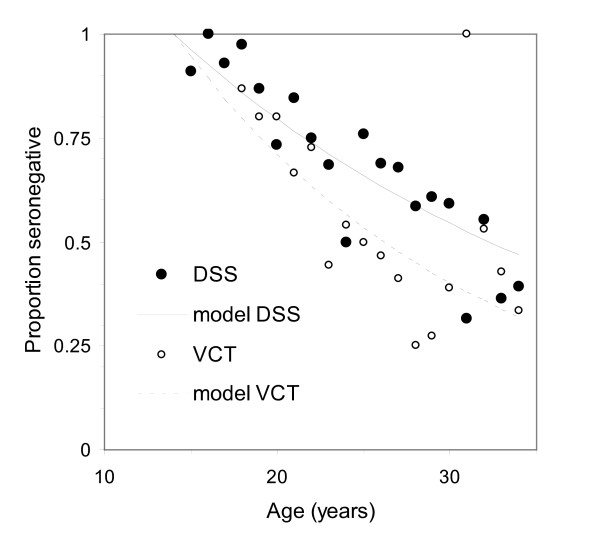
**Age-stratified proportions HSV-2 seronegative by age (years) and by study group for semi-urban women from Kilifi, coastal Kenya**. Data are shown for a random sample from the demographic surveillance system, DSS (filled marker) and a sample attending the VCT clinic (open marker). The corresponding best fit catalytic model curves (solid for DSS and dashed line VCT) are based on maximum log likelihood estimates (MLE) of the HSV-2 force of infection ( 95% CL, MLE) of 3.8% per annum (3.3-4.4, -319.532) in DSS women and 5.7% per annum (4.7-6.8, -168.233) in VCT women respectively.

The proportions of HSV-2 seronegative women recruited from the DSS declined from 94% in those aged 15-19 years to 47% in those aged >30 years. The corresponding proportions for VCT-women were 83% and 48%. In 30 DSS-women aged 15-16 years only 3% were HSV-2 seropositive. The fit of the maximum likelihood catalytic models to proportions seronegative by age and by source of recruitment (DSS or VCT) are shown in Figure 1. The estimated incidence of HSV-2 infection was 3.8 (95% CI: 3.3-4.4) per 100 seronegative women per year for DSS-participants (Table 4) and 5.7% (95% CI 4.7-6.8) per year for VCT participants.

**Table 4:** The estimated incidence of maternal and neonatal HSV-2 transmission in a random sample of 563 resident semi-urban women from Kilifi, Kenya

	Force of infection(/100 susceptible^$ ^women per year)	Estimated proportion susceptible (%)	Incidence of maternal infection (/1000 pregnancy per year)	Incidence of neonatal transmission (/100,000 births per year)
Estimate	3.8	58.6	17.1	33
LCL*	3.3	54.4	15.7	31
UCL	4.4	62.8	18.3	36

*LCL, UCL Lower and upper 95% confidence interval, respectively

^$ ^It is assumed that seronegative status equates with susceptibility

Using estimates of the force of infection acting on women in the DSS, we estimate an average proportion seronegative of 58.6% (95% CI: 54.4-62.8) over the child-bearing age. Hence the estimated incidence of maternal infections derived from Equation 3 is 17.1 (95% CI: 15.7-18.3) cases per 1,000 pregnancies per year, and the estimated incidence of neonatal transmission derived from Equation 4 is 33 (95% CI: 31-36) per 100,000 pregnancies per year (Table 4).

## Discussion

In this study we describe the serological epidemiology of prevalent HSV-2 infection among women of the semi-urban population of Kilifi, coastal Kenya, who were presumed to be either a relatively low risk group for HIV infection randomly selected from within a population under demographic surveillance or a self-selected presumed higher risk group for HIV infection recruited from a VCT-centre. Our aims were to characterize predictors of prevalent HSV-2 infection, estimate incidence of maternal HSV-2 infection within this population, and hence, indirectly, estimate the potential neonatal disease burden.

Overall, HSV-2 prevalence in DSS-women and VCT-women aged 15-34 years, at 32%, and 44%, respectively, is in the same range of the 42% national prevalence reported among Kenyan women from 15-64 years of age [19]. Similar to other studies, we found a rapid rise in HSV-2 seroprevalence from mid-late teenage years upwards coincident with onset of sexual activity [29]. Consistent with results of the national survey[19], we observed that HSV-2 prevalence increases with age. Analysis of factors associated with prevalent HSV-2 infection suggests a small but significantly higher proportion of women attending VCT with HSV-2 compared with the general population women selected through the DSS. However, the nature of the factors associated with prevalent HSV-2 infection in multivariate analysis appeared largely the same between the two groups.

Positive HIV-1 status was the strongest independent predictor of HSV-2 seropositivity, as reported elsewhere [29,30]. HIV-1 infection increases the risk of acquisition of HSV-2, the frequency and severity and reactivations [31,32]. Other variables associated with sexually transmitted diseases were also independently associated, but less strongly, with HSV-2 infection, including the presence of genital ulcers and having had a casual partner in the preceding 12 months.

Education beyond primary school (greater than year 8 in Kenyan schools), with a prevalence of 45% in this sample, was found to be inversely associated with HSV-2, with ~1.5 fold reduced likelihood of seropositivity. Similarly, a separate study has found that not finishing primary school was a significant predictor of HSV-2 prevalence among 469 women enrolled for an at-risk cohort of HIV-1 infection from nearby coastal areas [33]. Although education attainment has been inconsistently associated with HSV-2 sero-prevalence in prior studies [29,34,35], knowledge of HSV-2 infection among women in similar parts of East Africa has been very low [36]and the development of reproductive education programs, within the school curriculum, to increase awareness of HSV-2, is an intervention activity worth undertaking in Kenyan schools.

Using data from our prevalence study, we estimated the incidence of vertical transmission of HSV-2. The catalytic model we used was computed on the assumptions that HSV-2 shedding during delivery as a result of primary HSV-2 infection has a 50% risk of neonatal HSV-2 acquisition [5,15], and the risk of a baby acquiring HSV-2 from an infected mother occurs at primary infection within 11 days [28] (the average duration of viral shedding) of delivery [3,6,37]. Kilifi has a birth rate of 8000 live births per year [21], hence it is estimated that about 3 cases of neonatal herpes will occur per year among pregnant women in this population.

Earlier studies in Kilifi, assumed that the large number of perinatal deaths, especially those occurring at home, where cause could not be established, may have been related to HIV and sexually transmitted infections [21,38]. However, our data suggests that the burden of neonatal HSV-2 disease from maternal transmission is low and contributes insignificantly to the observed neonatal deaths in this region.

Interestingly, our estimate of the incidence of neonatal herpes is similar to the estimate of 1 case per 3200 live births from the USA [3]. However, most births in Kilifi district occur at home and options for appropriate interventions while mothers experience acute HSV-2 infections prior to birth are very limited. Where possible, prevention programmes aiming to reduce the burden of HIV-1 infections in women need to include information on HSV-2 infection. Pregnant women should be counselled to use condoms and avoid unprotected oral-genital contact during the last trimester of pregnancy and be advised to report to a referral health facility when signs of genital herpes infection are noted prior to delivery [14].

There are several limitations to this study. First, during the 2004 HIV-1 survey, records of women refusing participation in the HIV-1 survey were not kept. Therefore, we were unable to establish whether individuals who refused and those who participated in the study differed in characteristics. Second, this was a cross sectional study restricted to women only. Hence, the analysis of predictors of prevalent HSV-2 infection only established associations and could not make causal inferences. Third, we did not conduct any biological measurements of other common sexually transmitted diseases (e.g. syphilis) or perform visual inspection for the presence of genital ulcers. Lastly, we based our catalytic model on assumptions that HSV-2 incidence is independent of age (or time), even though, the risk for HSV-2 infection increases with age. In this study, including age-dependence in the rate of infection did not significantly improve the model fit. Furthermore, the data appear to follow a linear cumulative hazard from which we deduced that there was no good evidence for age (or time) dependence in HSV-2 incidence. We also, may have underestimated the burden of neonatal herpes infection as HSV-2 acquisition due to reactivated HSV-2 infections were not included in the model (considered to have less than 5% risk of neonatal herpes infections transmission), and HSV-1 infections in this study population were not measured.

## Conclusions

In summary, the existing burden of HSV-2 infection in the Kilifi semi-rural population is similar to national HSV-2 prevalence estimates in Kenya. HSV-2 transmission is rapid following the onset of sexual activity. Women infected with HSV-2 have an increased risk of acquiring HIV-1, and HSV-2 infection may remain a driving force behind the HIV-1 epidemic in this semi-urban population. Mother to child herpes transmission is unlikely to be a significant public health problem in this population. Educating young women about HSV-2 infection may help in reducing its burden in this semi-urban population.

## Competing interests

The authors declare that they have no competing interests.

## Authors' contributions

JUN designed, implemented the study, performed initial analysis and drafted the manuscript; EJS designed the HIV-1 study and revised the manuscript; CN performed HSV-2 laboratory testing and revised the manuscript; SW collected samples, conducted VCT counselling and revised the manuscript; KA performed HSV-2 laboratory testing and revised the manuscript; EB Revised the manuscript; MAP designed the HIV-1 study and revised the manuscript; PNA designed the study and revised the manuscript; DJN performed data analysis and revised the manuscript. All authors read and approved the final manuscript.

## Pre-publication history

The pre-publication history for this paper can be accessed here:

http://www.biomedcentral.com/1471-2334/11/155/prepub
